# Towards patient-specific prediction of conduction abnormalities induced by transcatheter aortic valve implantation: a combined mechanistic modelling and machine learning approach

**DOI:** 10.1093/ehjdh/ztab063

**Published:** 2021-08-20

**Authors:** Valeria Galli, Filip Loncaric, Giorgia Rocatello, Patricio Astudillo, Laura Sanchis, Ander Regueiro, Ole De Backer, Martin Swaans, Johan Bosmans, Joana Maria Ribeiro, Pablo Lamata, Marta Sitges, Peter de Jaegere, Peter Mortier

**Affiliations:** 1 FEops NV, Technologiepark 122, 9052 Ghent, Belgium; 2 Institute of Biomedical Research August Pi Sunyer (IDIBAPS), Carrer del Rosselló, 149, 08036, Barcelona, Spain; 3 Cardiovascular Institute, Hospital Clínic and Universitat de Barcelona, C. de Villarroel, 170, 08036 Barcelona, Spain; 4 Department of Cardiology, Rigshospitalet University Hospital, Blegdamsvej 9, 2100 København, Denmark; 5 Department of Cardiology, St. Antonius Ziekenhuis, Koekoekslaan 1, 3435 CM Nieuwegein, The Netherlands; 6 Department of Cardiology, University Hospital Antwerp, Drie Eikenstraat 655, 2650 Edegem, Antwerp, Belgium; 7 Department of Cardiology, Erasmus MC, Doctor Molewaterplein 40, 3015 GD Rotterdam, The Netherlands; 8 Department of Biomedical Engineering, King’s College London, Strand, London WC2R 2LS, UK; 9 Centro de Investigación Biomédica en Red de Enfermedades Cardiovasculares (CIBERCV), Instituto de Salud Carlos III, Av. Monforte de Lemos, 3-5, Pabellón 11, Planta 0 28029 Madrid, Spain

**Keywords:** TAVI, Conduction abnormalities, Machine learning, Mechanistic modelling, Digital twin

## Abstract

**Aims:**

Post-procedure conduction abnormalities (CA) remain a common complication of transcatheter aortic valve implantation (TAVI), highlighting the need for personalized prediction models. We used machine learning (ML), integrating statistical and mechanistic modelling to provide a patient-specific estimation of the probability of developing CA after TAVI.

**Methods and results:**

The cohort consisted of 151 patients with normal conduction and no pacemaker at baseline who underwent TAVI in nine European centres. Devices included CoreValve, Evolut R, Evolut PRO, and Lotus. Preoperative multi-slice computed tomography was performed. Virtual valve implantation with patient-specific computer modelling and simulation (CM&S) allowed calculation of valve-induced contact pressure on the anatomy. The primary composite outcome was new onset left or right bundle branch block or permanent pacemaker implantation (PPI) before discharge. A supervised ML approach was applied with eight models predicting CA based on anatomical, procedural and mechanistic data. CA occurred in 59% of patients (*n* = 89), more often after mechanical than first or second generation self-expanding valves (68% vs. 60% vs. 41%). CM&S revealed significantly higher contact pressure and contact pressure index in patients with CA. The best model achieved 83% accuracy (area under the curve 0.84) and sensitivity, specificity, positive predictive value, negative predictive value, and F1-score of 100%, 62%, 76%, 100%, and 82%.

**Conclusion:**

ML, integrating statistical and mechanistic modelling, achieved an accurate prediction of CA after TAVI. This study demonstrates the potential of a synergetic approach for personalizing procedure planning, allowing selection of the optimal device and implantation strategy, avoiding new CA and/or PPI.

## Graphical Abstract

**Figure:**
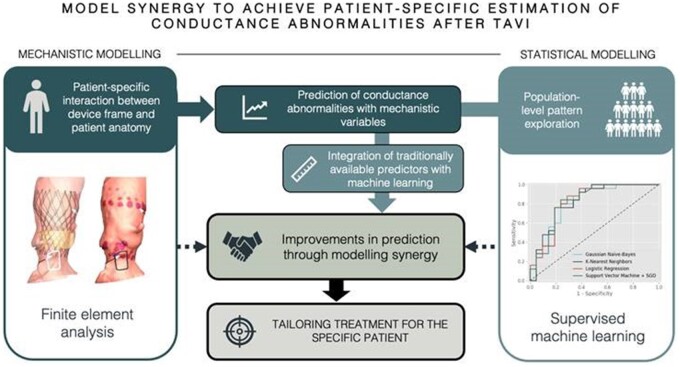

## Introduction

Transcatheter aortic valve implantation (TAVI) benefits patients with aortic stenosis at high, intermediate, and low surgical risk.^1–6^ Notwithstanding continuous improvements in outcomes due to advancement in catheter and valve technologies, as well as increase in experience, the occurrence of new conduction abnormalities (CA) and, consequently, new permanent pacemaker implantation (PPI) remains a clinical problem. The occurrence of new onset left bundle branch block with first generation valves was reported to vary between 4% and 65%.^7^ Acknowledging institution-dependent variables affecting PPI, an incidence varying between 2% and 51% was observed in a meta-analysis of 41 studies.^8^ Patient, procedure/operator, and device related factors have been shown to help estimate the risk of a new CA after TAVI on a population level, but the contribution of each factor in the individual patient remains unknown, highlighting the need for a more personalized approach in routine planning.

**Figure 1:**
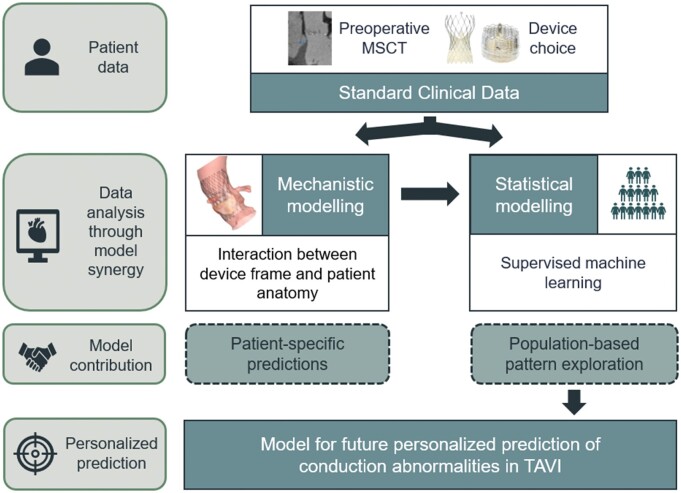
A personalized approach to risk stratification in TAVI planning using the combined synergy of mechanistic and statistical modelling.

Artificial intelligence is increasingly used to enhance the quality and efficacy of the planning, execution and evaluation of complex cardiac interventions such as TAVI.^9^ In parallel, developments in mechanistic models allows the addition of physiology and physics for a more profound identification of the mechanisms of cardiac disease and complications following medical interventions.^10^ Recently, the synergy between statistical and mechanistic models, combining inductive and deductive reasoning, has been suggested for the realization of precision medicine—more accurate diagnosis, treatment decision-making and, hence, prognosis.^11^ In this context, the entanglement of the mechanisms leading to CA after TAVI presents an appropriate challenge for such a tailored approach (*Figure 1*). The aim of this study was to develop and evaluate a patient-specific model predicting new CA after TAVI using machine learning (ML), combining statistical and mechanistic modelling.

## Methods

### Patient cohort

The retrospective index cohort consisted of 189 patients who had undergone TAVI in nine European centres. Patients with abnormal ECG [left/right bundle branch block (L/RBBB)] and/or permanent pacemaker before TAVI (*n* = 38) were excluded. Therefore, the final study cohort included 151 patients who were treated with the self-expanding CoreValve, Evolut R, and Evolut PRO valves (*n* = 107, Medtronic, MN, USA), or the mechanically expanding Lotus valve (*n* = 44, Boston Scientific, MA, USA). All patients were discussed in the multidisciplinary heart team. Valve type selection was based upon discretion of the operator/institution, whereas valve sizing was based upon multi-slice computed tomography (MSCT) derived baseline aortic root anatomy using the manufacturing sizing matrix, as described before.^12^ The primary and dependent outcome of interest was the occurrence of a new L/RBBB and/or PPI before discharge. All patients provided written informed consent for TAVI and the use of anonymous clinical, procedural, and follow-up data for research. The study was executed according to the Declaration of Helsinki, was approved by the ethics committees of all participating centres and did not fall under the scope of the Medical Research Involving Human Subjects Act.

### Anatomical measurements and patient-specific mechanistic modelling

**Figure 2:**
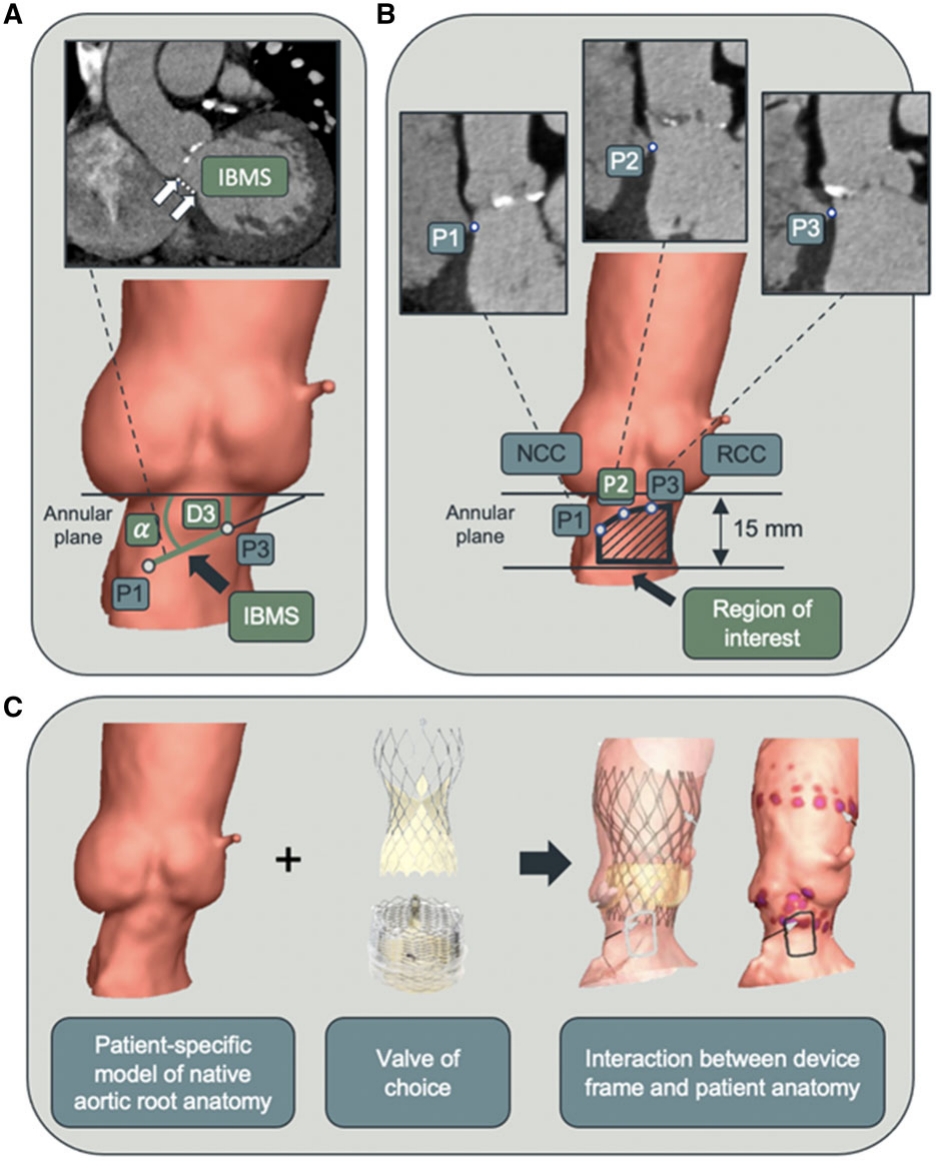
Identification of anatomical landmarks and mechanistic modelling workflow. (*A*) Identification of the inferior border of the membranous septum (IBMS) using preoperative MSCT images. P1 and P3 are landmarks selected at the beginning and end of the IBMS (white arrows), whereas P2 is an additional point in between to better track the course of the IBMS. The length of IBMS is calculated as the distance from P1 to P3. The angle between the segment connecting P1 and P3 and the annular plane is marked with α. The distance of P3 from the annular plane is marked with D3. (*B*) The region of interest for contact pressure analysis is defined by the area between the IBMS (extended towards the RCC by a 25° angle^12^) and the plane 15 mm below the annular level, to ensure inclusion of the proximal part of the left bundle branch. (*C*) A scheme showing the workflow of mechanistic modelling. A valve with known frame dimensions and mechanical properties is selected and virtually implanted into the patient-specific aortic root anatomy reconstructed from MSCT images. The device-host interactions are assessed—including aortic wall deformation and the resulting contact pressure exerted by the frame on the surrounding anatomy. The figure is based on ref.^12^ MSCT, multi-slice computed tomography; NCC, non-coronary cusp; RCC, right coronary cusp.

Preoperative MSCT was used for procedure planning and simulation. In-plane and through-plane resolution ranged from 0.31 to 0.97 mm/pixel, slice increment from 0.25 to 0.8 mm, and slice thickness from 0.5 to 1.5 mm. For the purpose of simulation, the MSCT of every patient was assessed and analysed by an independent simulation expert (FEops, Gent, Belgium). Some patients were excluded based on the following criteria of MSCT image quality: insufficient contrast, substantial movement in the aortic root and streaking artefacts, low planar scanning resolution, MSCT slice thickness higher than 1.5 mm, or slice increment higher than 0.9 mm. Furthermore, for this specific study, it was necessary to identify the membranous septum, thus patients whose images did not allow it were excluded. Quantification of the base of the aortic root (i.e. annulus and left ventricular outflow tract) including the inferior border of the membranous septum (IBMS) length, IBMS angulation with respect to the annular plane, and the shortest distance of the membranous septum (P3) to the annular plane were performed as previously described (*Figure 2A* and ref.^12^) The valve sizing index was calculated as the ratio between the nominal device diameter and the perimeter-derived annular diameter. Post-implantation angiograms were analysed to measure the depth of implantation (DOI)—defined as the average of the distance between the proximal (i.e. inflow) edge of the valve frame and the aortic annular plane on the non-coronary and the left coronary cusp side.^12^

A three-dimensional model of the aortic root was generated using MSCT. Finite element analysis was used to simulate valve implantation into the reconstructed anatomy: all steps of the actual implantation including the DOI were respected and the contact pressure exerted by the frame on the anatomy was computed.^12^^,^^13^ Of note, the segmented calcifications were included, thus accounting for their presence and specific location as well as mechanical properties. For the scope of the study (i.e. prediction of new CA), a region of interest (ROI) in which the conduction tissue is located was identified and consisted of the area defined by the IBMS and a plane 15 mm below the annular plane, inferiorly (*Figure 2B*). In this ROI, maximum contact pressure (Cpmax (MPa)) and contact pressure index (CPI, i.e. the percentage of the ROI subjected to contact pressure) were calculated (*Figure 2C* and ref.^12^). Further details of the modelling strategy are specified in Supplementary material online, *Methods*.

### Statistical modelling and machine learning

**Figure 3:**
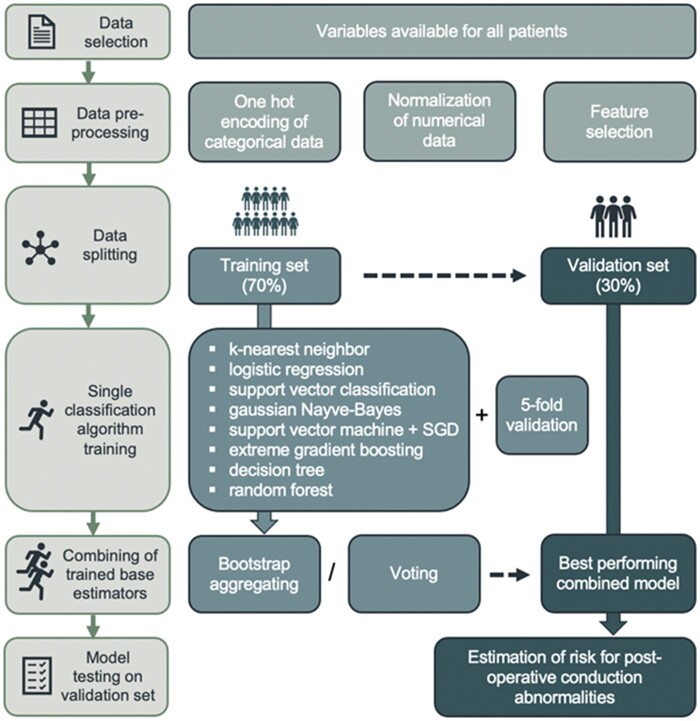
A scheme explaining data selection, preprocessing, supervised machine learning approach and post-processing.

Anatomical (*n* = 3; IBMS length, IBMS angulation, and the distance of P3 to the annular plane), procedural (*n* = 3; device type, sizing index, DOI), and mechanistic variables (*n* = 2; Cpmax, CPI) were included as the ML input. The analysis was conducted using the *scikit-learn* package (*v 0.20.4*) for *Python*. A scheme illustrating the central ML methodology is shown in *Figure 3*.

A preliminary exploratory data analysis was performed to investigate variable distributions (Supplementary material online, *Figure 1S*). Categorical variables (e.g. device type) were one hot encoded and numerical ones were normalized to a 0 to 1 scale as is common practice when data presents different value ranges. Additionally, feature (i.e. variable) selection was performed to search for highly correlated uninformative features: in this case, none of the eight features were correlated to another significantly and all were retained. After preprocessing, a supervised learning approach was applied: the cohort was split into training and validation sets (70–30%)—taking into account the distribution of categorical variables, that is, ensuring the presence of samples for each of the three devices in both sets. The training set was used to train the base classifiers (i.e. single classification algorithms) with five-fold cross-validation. We chose algorithms typically used for supervised binary classification, whose technical concepts have been described elsewhere^14^^,^^15^: (i) K-Nearest Neighbour Classifier, (ii) Logistic Regression, (iii) C-Support Vector Classification, (iv) Gaussian Naive-Bayes, (v) Support Vector Machine with Stochastic Gradient Descent (SGD) learning, (vi) Extreme Gradient Boosting Classifier, (vii) Decision Tree Classifier, and (viii) Random Forest Classifier. The trained base classifiers were then combined homogeneously (i.e. bootstrap aggregation) or in a mixed fashion (i.e. voting) and subsequently tested on the validation set. A more detailed explanation of the two ensembling techniques is available in the Supplementary material online. The optimal model was chosen based on accuracy and receiver operator characteristics (ROC) curves were considered with the corresponding area under the curve (AUC). On top of the usual statistics considered [sensitivity, specificity, positive predictive value (PPV), negative predictive value (NPV)], F1-score (harmonic mean of PPV and sensitivity) was taken into account as an indication of overall performance of the model.

To determine the importance of mechanistic variables in the prediction of CA, we evaluated their influence by testing the predictive power excluding Cpmax and CPI, as compared to the full model. Furthermore, using all variables, we assessed the performance for predicting L/RBBB only in a sub-cohort (*n* = 119), excluding patients who received a PPI after TAVI: in this case, the models were re-trained and the most accurate was selected.

### Statistics and outcome analysis

Continuous variables are expressed as mean ± standard deviation or median and interquartile range based on their distribution evaluated by the Shapiro–Wilk test. The categorical variables are expressed as number and percentage. Valve technology was differentiated into three categories: CoreValve, Evolut R/PRO, and Lotus—the former two being self-expanding and the latter mechanically expanding. Differences between groups were analysed for statistical significance with the Student’s *t*-test when comparing variables with normal distribution and the Mann–Whitney test for non-normally distributed variables. Contingency tables and a Chi-square test or Fisher’s exact test were used for categorical data.

## Results

### Cohort characteristics and patient-specific simulation results

**Figure 4:**
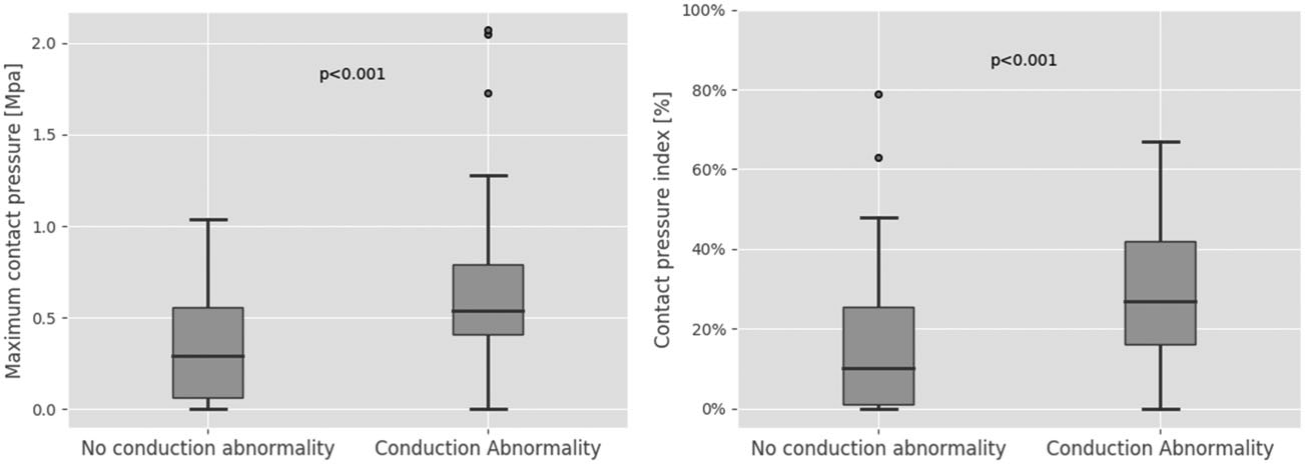
Results of the simulations stratified by occurrence of conduction abnormalities. Boxplots of maximum contact pressure (left) and contact pressure index (right). Numerical values are reported in *Table 2*.

Anatomical and procedural details are summarized in *Table 1*. The majority of patients received the first generation self-expanding CoreValve (55%), 16% received the second generation Evolut R/PRO. The mechanically expanding Lotus valve was used in 29%. A total of 89 patients (59%) developed new CA after TAVI, which was more frequent after mechanical (30/44, 68%) than after the first (50/83, 60%) or second generation (9/24, 37%) self-expanding valve implantation. Computer modelling and simulation (CM&S) revealed a significantly higher Cpmax (about two-fold median value) and CPI (about three-fold median value) in patients with new CA as compared to patients without (*Figure 4*, *Table 2*).

**Table 1:** Characteristics of the population

Parameter	All patients (*n* = 151)	CA, *N* = 89 (59%)	No CA, *N* = 62 (41%)	*P*-value
Anatomical				
Annular diameter,^a^ mm	24.06 ± 2.04	24.39 ± 2.10	23.60 ± 1.87	0.018
IBMS length, mm	10.07 ± 3.38	10.06 ± 3.64	10.09 ± 2.98	0.947
IBMS angle (α), °	19.55 ± 17.80	21.67 ± 16.91	16.50 ± 18.74	0.079
Depth of P3 (D3), mm	−2.10 ± 2.27	−1.87 ± 2.30	−2.44 ± 2.20	0.126
Pre-procedural				
Depth of implantation (DOI),^b^ mm	6.26 ± 4.13	7.30 ± 3.91	4.79 ± 4.02	<0.001
Device type				0.047
CoreValve (CV)	83 (55)	50 (56)	33 (53)	
Evolut R/PRO (ER/EPRO)	24 (16)	9 (10)	15 (24)	
Lotus (LT)	44 (29)	30 (34)	14 (23)	
Device size				0.462
CV 26	29 (20)	17 (20)	12 (21)	
CV 29	48 (32)	28 (31)	20 (32)	
CV 31	6 (4)	5 (6)	1 (2)	
ER/EPRO 26	9 (6)	2 (2)	7 (11)	
ER/EPRO 29	15 (8)	7 (4)	8 (13)	
LT 23	11 (7)	5 (6)	6 (10)	
LT 25	18 (12)	12 (13)	6 (10)	
LT 27	15 (10)	13 (15)	2 (3)	
Sizing index^c^	1.14 ± 0.10	1.12 ± 0.09	1.15 ± 0.10	0.081
Post-procedural				
RBBB	1 (0)	1 (1)	0 (0)	
LBBB	78 (52)	78 (88)	0 (0)	
PPI	32 (21)	32 (36)	0 (0)	

Values are given as mean ± standard deviation or *n* (%). Percentages refer to the total reported in the first row (e.g. *N* = 89 for the CA column).

IBMS, inferior border of membranous septum; LBBB, left bundle branch block; RBBB, right bundle branch block.

a Perimeter-based diameter = annular perimeter/π.

b Implantation depth assessed in postoperative angiograms: distance from the aortic annular plane on the NCC side to the deepest level of the most proximal edge of the device frame (NCC: non coronary cusp).

c Nominal device diameter/annular diameter.

**Table 2:** Computer modelling and simulation results

Parameter	All patients (*n* = 151)	CA, *n* = 89 (59%)	No CA, *n* = 62 (41%)	*P*-value
Maximum contact pressure (MPa)	0.45 (0.26–0.67)	0.55 (0.41–0.79)	0.30 (0.06–0.56)	<0.001
Contact pressure index (%)	22 (8–37)	28 (18–44)	10 (1–26)	<0.001

Values are given as median (interquartile range).

### Machine learning for prediction of conduction abnormalities

**Figure 5:**
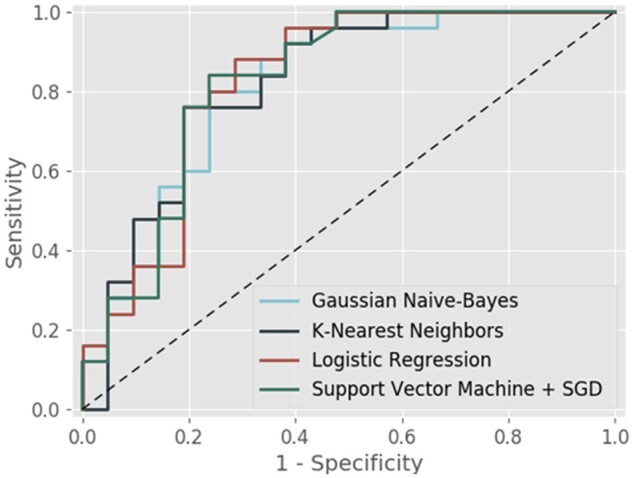
Receiver operator characteristic curves for the four most accurate algorithms.

The ensemble models were compared based on accuracy score: the homogeneous ensemble (bootstrap aggregation) of K-Nearest Neighbour showed the best performance with 83% accuracy and sensitivity, specificity, PPV, NPV, F1-score, and AUC of 100%, 62%, 76%, 100%, and 82% and 0.84, respectively (*Table 3*). The ROC curves of the four most accurate models are shown in *Figure 5*.

**Table 3:** Relevant statistics for the four most accurate algorithms

Model	Accuracy	Sensitivity	Specificity	PPV	NPV	F1-score	AUC (95% CI)
K-Nearest Neighbours	83%	1.00	0.62	0.76	1.00	0.82	0.84 (0.69–0.95)
Logistic Regression	80%	0.96	0.62	0.75	0.93	0.83	0.80 (0.70–0.96)
Support Vector Machine + SGD	78%	1.00	0.52	0.71	1.00	0.83	0.81 (0.69–0.95)
Gaussian Naive-Bayes	76%	0.84	0.67	0.75	0.78	0.82	0.77 (0.69–0.95)

AUC, area under curve; NPV, negative predictive value; PPV, positive predictive value.

A sharp decrease in accuracy was observed when excluding Cpmax and CPI as features, with the best ensemble (Support Vector Machine + SGD) having 67% accuracy and 0.72 AUC. The results for this sub analysis are reported in Supplementary material online, *Table 1S*.

The analysis of the prediction of only L/RBBB in the sub-cohort of patients of 119 who underwent TAVI and did not receive a new PPI after TAVI, revealed the highest accuracy when using the Support Vector Machine + SGD (75% with 0.77 AUC—Supplementary material online, *Table 2S*).

An overview of all the analyses and related performance is reported in Supplementary material online, *Figure 2S*.

## Discussion

In this multicentric study, we demonstrate the synergy of statistical and mechanistic modelling in predicting new CA after TAVI. The independent variables derived from patient-specific mechanistic modelling, reflecting the interaction between device and host (i.e. contact pressure), were highly predictive of CA. Also, ML-based statistical modelling helped to enhance the predictive ability through the integration of these mechanistic markers with the traditional patient and procedure-related predictive variables. Noteworthy, the prediction ability of the herein proposed model was not only true for the composite of new L/RBBB and new PPI (primary objective of study) but also for new L/RBBB as single endpoint.

The findings of this study need to be interpreted in the light of sample size, patient demographics and valve technology. The cohort of 151 patients was predominantly treated with the first generation self-expanding (55%) or the recently withdrawn mechanical expanding valve (29%). Yet, it goes without saying that the proposed model can in principle be applied to any valve technology. New CA (i.e. L/RBBB and new PPI) occurred in 89 out of the 151 patients (59%). The frequency of new CA after the implantation of the first generation self-expanding CoreValve and the mechanically expanding Lotus valve is in accordance with previously reported clinical observations.^8^^,^^12^^,^^16^ This also holds for the Evolut platform of which the Evolut R and Evolut PRO only differ in sealing skirt (affecting paravalvular leak) but not in design and mechanical behaviour.^17^^,^^18^

ML-based prediction of new CA and/or PPI is subject to recent research with varying degrees of performance. Gomes et al reported an AUC of 0.61 for ML-based prediction of new PPI after TAVI.^19^ Similarly, low accuracy of PPI prediction was seen in a gradient boosting model incorporating 141 heterogeneous demographic, clinical, ECG, and imaging variables (AUC 0.66).^20^ A performance comparable to our model, was achieved by Truong *et al*.^21^ using a random forest algorithm incorporating baseline ECG, device-related and imaging variables (AUC 0.88). Reasons for disparity in performance in this field of ML-based prediction modelling are multifold. A high-quality dataset consisting of sufficient heterogeneous/multidimensional data as well as the selection of relevant variables are important for model performance. Nevertheless, the generation of novel variables, such as mechanistic ones—as shown in this study—may enhance the predictive power, reduce the number of variables needed and thus increase clinical applicability.

### Predicting conduction abnormalities through the synergy of statistical and mechanistic modelling

Patient-specific CM&S allows the assessment of the interaction between the device and host, generating mechanistic variables that incorporate all the factors playing a role in the injury to the conduction tissue—such as calcifications, sizing, etc. These variables have shown to be predictive of new CA after TAVI.^12^^,^^13^^,^^17^ It is clear that the addition of mechanistic parameters to the model comes with the computational cost, but also increases accuracy. In fact, excluding those from the primary model, the predictive power decreased (Supplementary material online, *Table 1S*).

Statistical modelling, through ML or traditional statistical classification algorithms, can improve the prediction of mechanistic variables by combining those with other, traditionally available parameters. In classification tasks such as prediction of adverse events, the selection of tools used in traditional statistical modelling is expanded by ML algorithms, enabling to find the optimal model for the dataset at hand.^22^ In our study, predictors of CA were: descriptors of the aortic valve and ascending aorta (i.e. the shortest distance of the membranous septum to the annular plane (P3), length and angulation of IBMS (length of IBMS was previously observed as strong predictor of atrio-ventricular block^23^ and PPI^18^), procedure-related parameters (i.e. valve type, size and DOI), and mechanical properties of the implanted device.^24^^,^^25^ The added value of ML integration enhanced the observed predictive power based on mechanistic variables only, where statistical univariate and multivariate analyses on subsets of our cohort revealed accuracy of 76% and 77% for CPmax and CPI in CoreValve and Evolut R valves,^12^ and analogously 75% and 71% in a subset with Lotus valve alone.^13^

We believe that the synergy between statistical and mechanistic modelling helps the development of patient-tailored, personalized or precision medicine.^11^ Clinicians are becoming gradually familiar with ML-based approaches, yet, mechanistic modelling is still a niche concept in clinical medicine. Mechanistic models allow the extraction and combination of patient-specific biomarkers that stem from computer simulations such as herein reported (i.e. patient-specific interaction of valve with MSCT derived anatomy). The value of such a combined approach has—among others—been demonstrated in cardiac resynchronization therapy^26^ and hypertrophic cardiomyopathy.^27^ In case of aortic valve disease, such an approach can be applied prospectively in order to provide a tailored prediction of adverse events and, thus, improve patient selection, procedure planning and execution by modelling all relevant parameters before the procedure (device type and size as well as DOI).

### Limitations

The presented model stems from a medium-sized cohort from nine European centres. The number of variables included in the analysis is limited by the retrievability of data. Information on demographics, comorbidities, baseline clinical variables, and echocardiograms was not available for the complete cohort and were, therefore, not used as additional (candidate) predictors in the modelling. Similarly, the exact timing of L/RBBB insurgence or PPI—periprocedurally or after discharge—was not available. On the other hand, data from MSCT imaging, with detailed anatomical descriptors of the aortic valve and ascending aorta, was paired with case-specific mechanistic modelling. Validation of the model using an external data set has not been performed.

The composite outcome of L/RBBB and PPI is hindered by the variability in clinical decision-making of PPI between and within centres. Therefore, we performed an additional analysis to predict the outcome of L/RBBB as the only independent outcome-measure in the sub-cohort of patients who underwent TAVI free from new PPI after procedure, showing slightly lower accuracy when applying this more robust endpoint.

As far as the computational modelling is concerned, although it would be more appropriate to consider distributions of the mechanistic parameters at varying implantation depths and device rotation, this would be computationally too expensive, especially in the perspective of a larger dataset.

In order to confirm the generalizability of our findings, a more comprehensive dataset featuring additional parameters such as age, gender, body surface area, and other common clinical variables will be needed.

Lastly and as mentioned above, most patients were treated with the first generation CoreValve and the recently withdrawn mechanical-expanding Lotus valve. Few patients were treated with currently used self-expanding valves. Balloon-expandable valves were not included. Yet, the model herein proposed does not depend on the valve technology itself but can be applied to any commercial device.

## Conclusions

Combining statistical and mechanistic modelling helps the development of patient-specific prediction of adverse events after TAVI. Through data integration with traditional predictors of CA, ML-based classification models enhance the predictive ability of patient-specific mechanistic modelling. We believe that application of the presented method applied on prospective patients in the future may help the physician to select the device that best fits the individual patient and implantation strategy.

## Lead author biography

**Figure:**
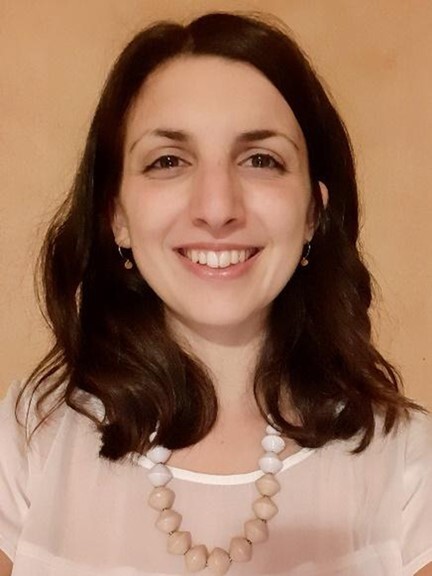


Valeria Galli holds a Master of Science (summa cum laude) in Biomedical Engineering obtained at Politecnico di Milano. She currently works as R&D engineer at FEops nv (Gent, Belgium) while being Early-Stage Researcher in the EU H2020 project ‘Personalised In-Silico Cardiology’. She is active in the field of patient-specific computer simulations for structural heart interventions (finite element analysis and computational fluid dynamics). Her interests include the application of artificial intelligence methods for prediction of procedural outcome, automated image segmentation, and landmarks detection.

## Supplementary material

Supplementary material is available at *European Heart Journal – Digital Health* online.

**Consent:** All patients provided written informed consent for TAVI and the use of anonymous clinical, procedural, and follow-up data for research.

## Funding

This work was supported by Horizon 2020 European Commission Project H2020-MSCA-ITN-2016 (764738). P.L. holds a Wellcome Trust Senior Research Fellowship (209450/Z/17/Z).

**Conflict of interest:** V.G., G.R., and P.A. are employees of Feops. P.M. is shareholder of FEops. All other co-authors declared that they have no conflict of interest. Given his role as deputy editor, P.d.J. had no involvement in the peer review of this article and has no access to information regarding its peer review. Full responsibility for the editorial process for this article was delegated to Joost Lumens.

## Data availability

The data underlying this article cannot be shared publicly due to the privacy of individuals that participated in the study and for reasons of intellectual property.

## Supplementary Material

ztab063_Supplementary_Data
