# Supplementary material for: Towards patient-specific prediction of conduction abnormalities induced by transcatheter aortic valve implantation: a combined mechanistic modelling and machine learning approach
Source: Eur Heart J Digit Health. 2021 Aug 20;2(4):606–15. doi: 10.1093/ehjdh/ztab063 (PMC9708019; doi:10.1093/ehjdh/ztab063)
Supplement: ztab063_Supplementary_Data [file ztab063_supplementary_data.docx]

**Supplementary materials**

**Patient specific computer simulations**

For all patients, computer simulations were run with device, device size and depth of implantation as requested by the clinician prior to the intervention. Detailed explanation of the model validation and tuning are available in previous studies.[(1,2)](https://paperpile.com/c/jTJgvw/Y6gJ+GqgY) Among others, assumptions were made to build a relatively simple but realistic model of the geometries (device, aortic root and aortic valve leaflets). More specifically, a linear elastic material was deemed acceptable based on previous findings which reported comparable accuracy in terms of frame deformation when using a simple linear elastic material as compared to a more complex hyperelastic material.[(3,4](https://paperpile.com/c/jTJgvw/3Itf+qE5T)) Also, the thickness of the heart tissue should be ideally patient specific, measured and tuned for each patient, however this is objectively difficult to assess in vivo, and hence, unfeasible. Therefore, a constant thickness of 1mm for the aorta and 1.5mm for the leaflets was assumed in our model. Anatomic studies showed that the thickness of the aortic leaflets decreases towards the free edge. Based on the data reported by Grande et al.[(5)](https://paperpile.com/c/jTJgvw/YJSL) and Sahasakul et al.[(6)](https://paperpile.com/c/jTJgvw/TdkV) the valve thickness increases significantly with age - up to 1.42 mm in patients older than 60 years. Therefore, we believe that the value we adopted is representative for the elderly population included in this study. The assumptions used in our models have previously resulted in accurate simulation results in terms of frame deformation and calcium displacement, as well as predicted paravalvular regurgitation.[(2,7)](https://paperpile.com/c/jTJgvw/aKcE+GqgY)

The elastic modulus, thickness, spring stiffness and coefficient of friction were all included in the back calculation/calibration analysis performed manually. Material parameters and thickness were investigated first; friction was then included to capture the non-uniform strut distribution of the device frame as observed on post-operative MSCT. Normally, one (or two parameters) were (simultaneously) modified at each iteration, based on the evaluation of the Finite Element Analysis result. The analysis consisted in more than 30 iterations.

**Ensemble Methods**

Homogeneous and heterogeneous ensembling were used to enhance the power of the single base classifiers (i.e., K-Nearest Neighbor, Logistic Regression, etc): the aim is to achieve better performance by reducing either bias or variance. Specifically, bootstrap aggregation was used to combine multiple classifiers of the same type (e.g., K-Nearest Neighbor + K-Nearest Neighbor ...), whereas voting was used to mix different based classifiers (e.g., K-Nearest Neighbor + Logistic Regression + Gaussian Naive-Bayes ...).

The chart below elucidates the basic mechanisms of bagging: the same classifier is applied to different samples taken from the training and their predictions are averaged to obtain the final prediction.


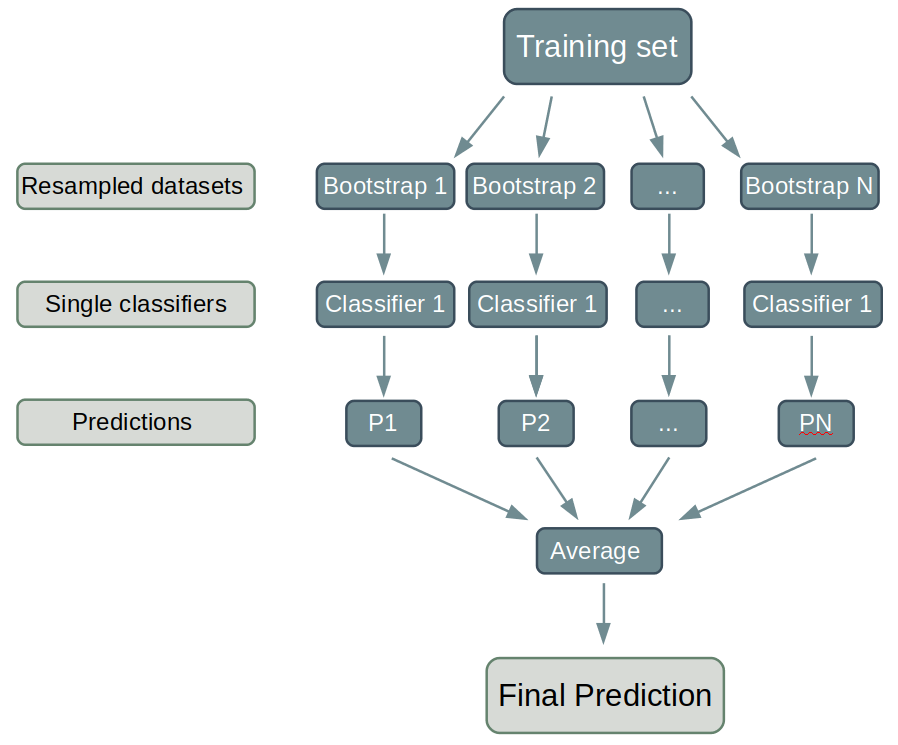


On the other hand, in voting ensembles different classifiers are applied to the same training set and their predictions are averaged to obtain the final prediction (soft voting).


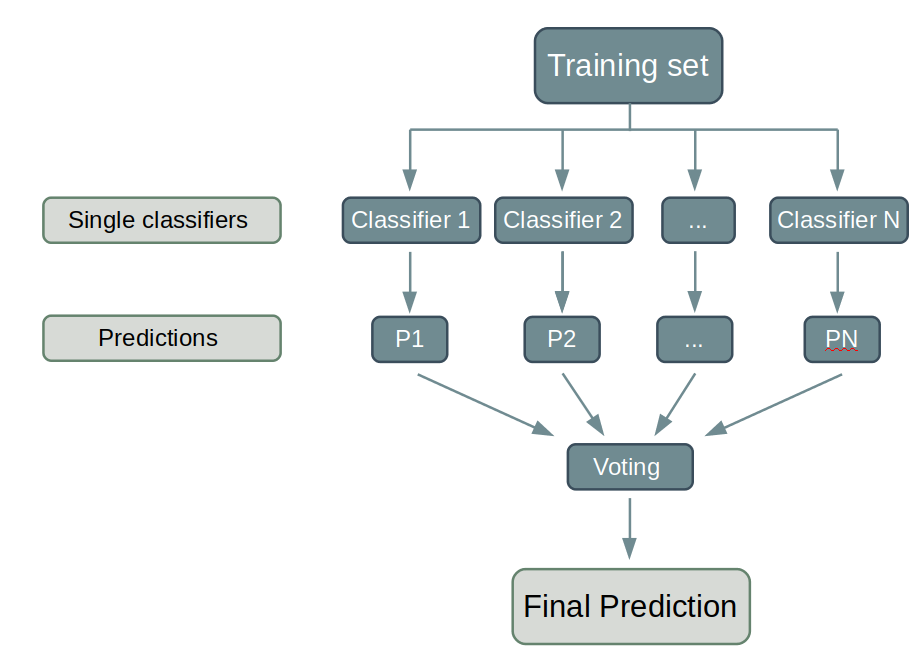


**Supplementary Tables**

**Table S1: Results for the prediction on the full cohort (n=151) excluding the mechanistic features Cpmax and CPI.**

| **Model** | **Accuracy** | **Sensitivity** | **Specificity** | **PPV** | **NPV** | **F1-score** | **AUC**  **[95% CI]** |
| --- | --- | --- | --- | --- | --- | --- | --- |
| Support Vector Machine + SGD | 67% | 0.69 | 0.62 | 0.53 | 0.79 | 0.60 | 0.72 [0.54 – 0.90] |
| Logistic Regression | 64% | 0.77 | 0.62 | 0.50 | 0.81 | 0.61 | 0.69 [0.50 – 0.88] |
| Decision Tree Classification | 64% | 0.69 | 0.52 | 0.50 | 0.78 | 0.58 | 0.81 [0.52 – 0.88] |
| Support Vector Classification | 61% | 0.77 | 0.67 | 0.48 | 0.80 | 0.59 | 0.64 [0.47 – 0.84] |

PPV - Positive Predictive Value; NPV - Negative Predictive Value; AUC - area under curve.

**Table S2: Results for the prediction of L/RBBB (n=119).**

| **Model** | **Accuracy** | **Sensitivity** | **Specificity** | **PPV** | **NPV** | **F1-score** | **AUC**  **[95% CI]** |
| --- | --- | --- | --- | --- | --- | --- | --- |
| Support Vector Machine + SGD | 75% | 0.69 | 0.78 | 0.64 | 0.82 | 0.67 | 0.77 [0.61 – 0.93] |
| Random Forest | 72% | 0.85 | 0.65 | 0.58 | 0.88 | 0.69 | 0.75 [0.50 – 0.88] |
| Gaussian Naïve-Bayes | 69% | 0.85 | 0.61 | 0.55 | 0.88 | 0.67 | 0.77 [0.58 – 0.91] |
| Decision Tree Classification | 69% | 0.69 | 0.70 | 0.56 | 0.80 | 0.62 | 0.73 [0.61 – 0.94] |

PPV - Positive Predictive Value; NPV - Negative Predictive Value; AUC - area under curve.

**Supplementary Figures**

**Figure S1: Pair plot showing the relationship between all features color coded by the outcome.**


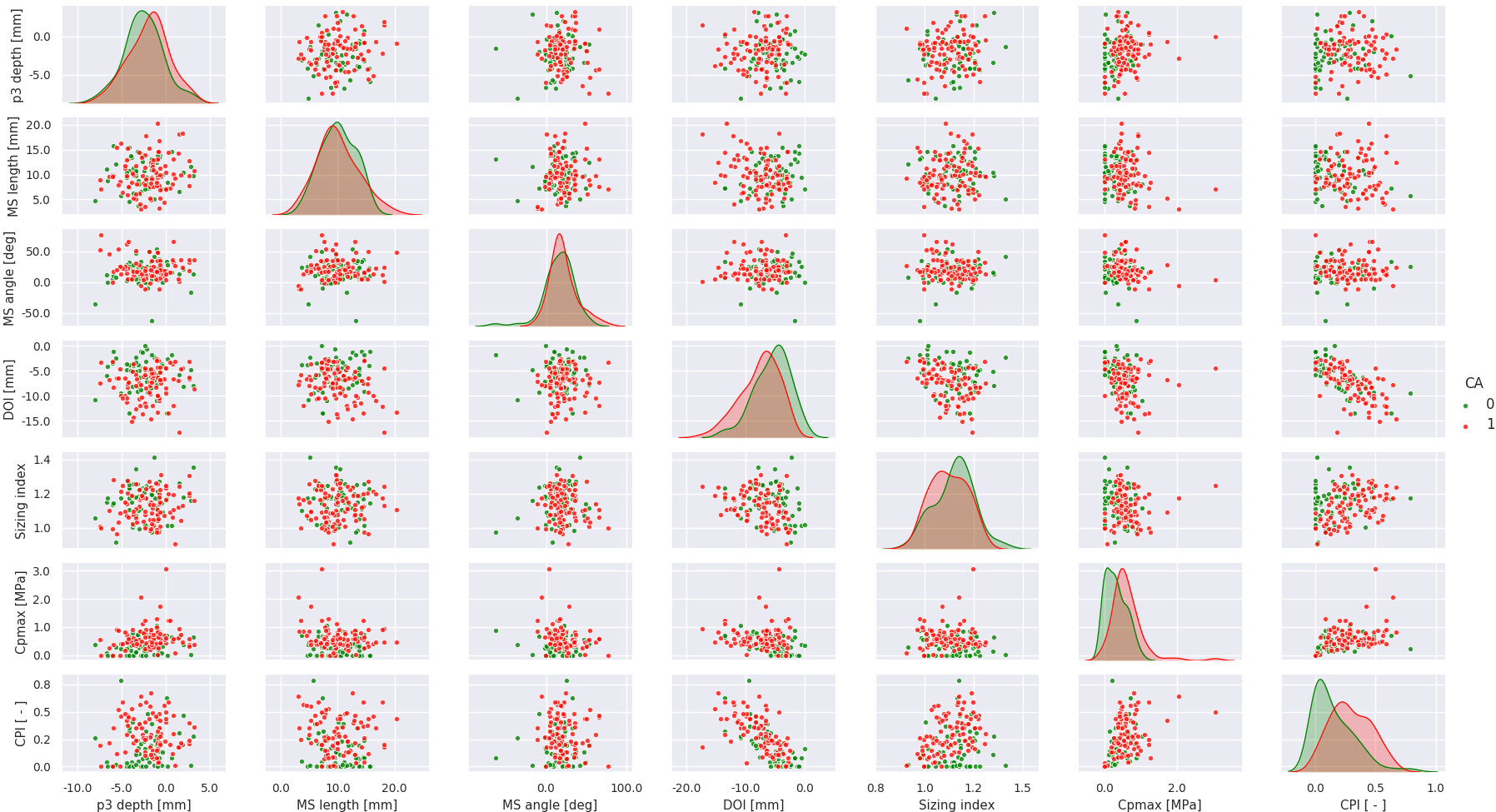


Univariate distributions along the diagonal highlight any visible shift between the two distributions (CA=0 vs CA=1) in single features (e.g., no visible shift for some variables but evident for Cpmax, CPI); scatterplots of bivariate relations show no recognizable pattern, overlapping of the groups, data dispersion and the presence of outliers.

**Figure S2: Overview of the analyses conducted on the whole cohort and on sub-cohorts with corresponding performance.**


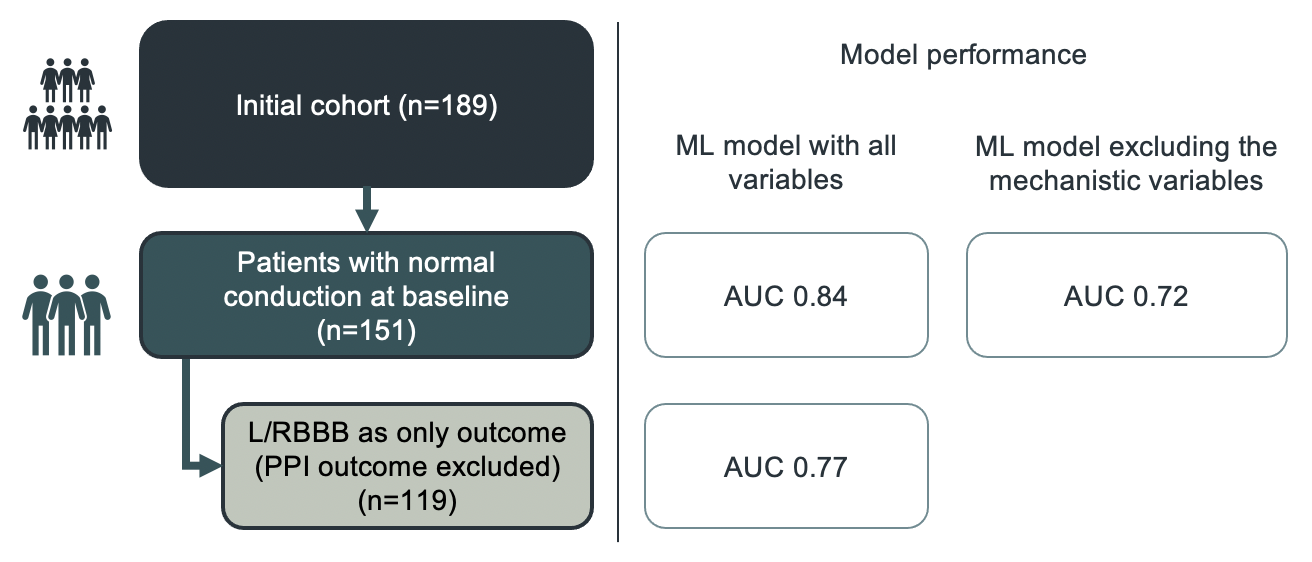


**References**

1. [Rocatello G, El Faquir N, De Santis G, Iannaccone F, Bosmans J, De Backer O, Sondergaard L, Segers P, De Beule M, de Jaegere P, Mortier P. Patient-Specific Computer Simulation to Elucidate the Role of Contact Pressure in the Development of New Conduction Abnormalities After Catheter-Based Implantation of a Self-Expanding Aortic Valve. Circ Cardiovasc Interv. 2018;**11**(2):e005344.](http://paperpile.com/b/jTJgvw/Y6gJ)

2. [Schultz C, Rodriguez-Olivares R, Bosmans J, Lefèvre T, De Santis G, Bruining N, Collas, V., Dezutter, T., Bosmans, B., Rahhab, Z., El Faquir, N., Watanabe, Y., Segers, P., Verhegghe, B., Chevalier, B., van Mieghem, N., De Beule, M., Mortier, P., de Jaegere, P. Patient-specific image-based computer simulation for the prediction of valve morphology and calcium displacement after TAVI with the Medtronic CoreValve and the Edwards SAPIEN valve. EuroIntervention. 2016;**11**:1044–52.](http://paperpile.com/b/jTJgvw/GqgY)

3. [Russ C, Hopf R, Hirsch S, Sündermann S, Falk V, Székely G, Gessat M. Simulation of transcatheter aortic valve implantation under consideration of leaflet calcification. Conf Proc IEEE Eng Med Biol Soc. 2013:711–4.](http://paperpile.com/b/jTJgvw/3Itf)

4. [Finotello A, Morganti S, Auricchio F. Finite element analysis of TAVI: Impact of native aortic root computational modeling strategies on simulation outcomes. Med Eng Phys. 2017;**47**:2–12.](http://paperpile.com/b/jTJgvw/qE5T)

5. [Grande-Allen KJ, Jane Grande-Allen K, Cochran RP, Reinhall PG, Kunzelman KS. Re-creation of sinuses is important for sparing the aortic valve: A finite element study. The Journal of Thoracic and Cardiovascular Surgery. 2000;**119**:753–63.](http://paperpile.com/b/jTJgvw/YJSL)

6. [Sahasakul Y, Edwards WD, Naessens JM, Tajik AJ. Age-related changes in aortic and mitral valve thickness: implications for two-dimensional echocardiography based on an autopsy study of 200 normal human hearts. Am J Cardiol. 1988;**62**(7):424–30.](http://paperpile.com/b/jTJgvw/TdkV)

7. [de Jaegere P, De Santis G, Rodriguez-Olivares R, Bosmans J, Bruining N, Dezutter T, Rahhab, Z., El Faquir, N., Collas, V., Bosmans, B., Verhegghe, B., Ren, C., Geleinse, M., Schultz, C., van Mieghem, N., De Beule, M., Mortier, P. Patient-Specific Computer Modeling to Predict Aortic Regurgitation After Transcatheter Aortic Valve Replacement. JACC Cardiovasc Interv. 2016;**9**(5):508–12.](http://paperpile.com/b/jTJgvw/aKcE)
